# Association of Waist Circumference and Body Composition Indices with Insulin Resistance in Prepubertal Children with Obesity

**DOI:** 10.3390/metabo16080569

**Published:** 2026-08-11

**Authors:** Seulki Kim, Moon Bae Ahn

**Affiliations:** 1 Department of Pediatrics, Eunpyeong St. Mary’s Hospital, College of Medicine, The Catholic University of Korea, Seoul 03312, Republic of Korea; seulki12633@gmail.com; 2 Division of Endocrinology, Department of Pediatrics, St. Nicholas Children’s Hospital, Seoul St. Mary’s Hospital, College of Medicine, The Catholic University of Korea, Seoul 06591, Republic of Korea

**Keywords:** pediatric obesity, insulin resistance, waist circumference, body composition

## Abstract

**Background**: Body mass index (BMI) is widely used to assess pediatric obesity but has limitations in distinguishing between fat and muscle mass. This study aimed to investigate the association between various body composition parameters and insulin resistance (IR) and to evaluate their clinical utility in prepubertal children with obesity. **Methods**: This retrospective cross-sectional study evaluated prepubertal children with obesity. Anthropometric measures and body composition, including percent body fat (PBF) and fat-to-muscle ratio (FMR), were assessed using bioelectrical impedance analysis. IR was defined as a homeostasis model assessment of insulin resistance (HOMA-IR) ≥2.5. Spearman rank correlation, linear regression, and multivariate logistic regression analysis were performed to identify independent associated factors for IR. **Results**: A total of 147 prepubertal children (61 males, 86 females) were analyzed. The IR group exhibited significantly higher waist circumference SDS (WC_SDS), waist circumference-to-height ratio SDS (WHtR_SDS), and FMR compared to the non-IR group (all *p* < 0.05). In correlation analysis, HOMA-IR showed significant positive associations with WC_SDS (ρ = 0.39) and FMR (ρ = 0.22). After adjusting for age and sex, WC SDS (odds ratio (OR) = 6.04; 95% CI: 2.47–14.75; *p* < 0.001), FMR (OR = 4.41; 95% CI: 1.16–16.78; *p* = 0.030), BMI SDS (OR = 2.31; 95% CI: 1.30–4.10; *p* = 0.004), and PBF (OR = 1.12; 95% CI: 1.04–1.20; *p* = 0.004) remained independent risk factors for IR. **Conclusions**: In prepubertal children with obesity, WC SDS, FMR, BMI SDS and PBF were each significantly associated with IR after adjusting for age and sex. These findings suggest that measures of central adiposity and fat-to-muscle balance, in addition to conventional weight-based indices, are relevant to metabolic risk assessment in this age group.

## 1. Introduction

The prevalence of childhood obesity has increased alarmingly worldwide, including in Korea, emerging as one of the most critical public health challenges of the 21st century [1,2]. Obesity in childhood is strongly associated with early-onset metabolic derangements, most notably insulin resistance (IR), dyslipidemia, and an increased risk of developing type 2 diabetes and cardiovascular diseases later in life [3,4]. Because IR constitutes the fundamental pathophysiological mechanism underlying metabolic syndrome and represents the earliest detectable metabolic consequence of excess adiposity [5], timely identification of high-risk children and implementation of targeted interventions are of paramount importance in pediatric practice [6].

Body mass index (BMI) or its age- and sex-standardized form, BMI standard deviation score (BMI_SDS), remains the most widely used screening tool for childhood obesity due to its simplicity and low cost [7]. However, BMI carries well-recognized limitations. Because it relies solely on body weight and height, it fundamentally fails to distinguish between fat mass and lean mass [8]. This inability to differentiate body compartments can lead to clinical misclassification in two directions. On one hand, individuals with high muscle mass but healthy fat levels can be erroneously mislabeled as overweight or obese. On the other hand, previous studies highlight that BMI’s diagnostic performance is limited by its low sensitivity, meaning it frequently overlooks children who possess excessive body fat despite not meeting the BMI criteria for obesity [9]. Furthermore, BMI cannot provide information regarding body fat distribution, meaning it cannot distinguish between relatively benign subcutaneous fat and highly diabetogenic visceral fat or ectopically deposited fat in organs such as the liver [7,8,10]. Thus, relying solely on BMI may lead to the misclassification of actual metabolic risk, particularly in growing children whose body composition changes dynamically [11].

To overcome the limitations of BMI, various anthropometric and body composition indices have been proposed. Waist circumference (WC) and waist-to-height ratio (WHtR) are well-established, practical surrogate markers for central adiposity, which is more directly linked to metabolic complications than generalized obesity [8,12,13,14,15].

Several novel indices have recently been proposed to refine metabolic risk assessments. The Body Roundness Index (BRI), derived from WC and height via an elliptical geometry model, more accurately estimates visceral adiposity than WHtR and has demonstrated superior predictive performance over BMI for cardiometabolic outcomes in children [16,17]. A Body Shape Index (ABSI; WC/[BMI^2^/^3^ × height^1^/^2^]) and the Cardiometabolic Index (CMI; WHtR × TG/HDL-C ratio) have each shown associations with metabolic risk in adults and, to a lesser extent, in obese children; however, pediatric-specific reference values remain unestablished, and CMI additionally requires fasting lipid measurements [18]. The triglyceride–glucose (TyG) index—calculated as the natural logarithm of [fasting triglycerides (mg/dL) × fasting glucose (mg/dL)/2]—offers a cost-effective biochemical surrogate of IR that avoids insulin assays, though its diagnostic accuracy is highly variable across pediatric studies and age- and pubertal-stage-specific cutoff values have not been standardized [19,20,21]. Importantly, all of these indices share a critical limitation: they do not capture the qualitative balance between fat mass and skeletal muscle, which has emerged as a key determinant of insulin sensitivity.

With the widespread clinical application of bioelectrical impedance analysis (BIA), body composition indices reflecting the balance between fat and muscle have gained considerable attention [10]. Percent body fat (PBF) directly quantified the proportion of fat mass, providing a more physiologically meaningful measure of adiposity than dimension-based indices. Among emerging parameters, the fat-to-muscle ratio (FMR), defined as fat mass divided by skeletal muscle mass, has gained considerable attention. Because skeletal muscle is the primary tissue responsible for insulin-mediated glucose disposal, a disproportionate accumulation of fat mass relative to muscle mass—reflected by a high FMR—may significantly exacerbate IR [8,22,23]. Prior studies in adults have confirmed that FMR is independently associated with IR and metabolic syndrome and that the fat-to-lean balance constitutes a critical determinant of insulin sensitivity [22,23]. In alignment with these insights, the 2025 Lancet Diabetes & Endocrinology Commission recommended incorporating body composition assessment beyond BMI for individualized obesity management and explicitly defined clinical obesity as excess adiposity directly causing organ dysfunction such as IR [14].

Despite the growing number of available indices, studies directly comparing the predictive utility of multiple parameters—spanning conventional anthropometrics (BMI-SDS, WC-SDS, and WHtR-SDS), novel composite indices, and BIA-derived body composition measures (PBF and FMR)—for IR within a well-characterized pediatric cohort remain scarce. Furthermore, puberty itself induces a physiological, transient state of IR attributable to growth hormone-mediated antagonism of insulin action, independent of adiposity changes [24]; evaluating these metabolic markers exclusively in prepubertal children is therefore essential to accurately assess the direct impact of obesity-related body composition on IR without pubertal confounding. Therefore, the present study aimed to investigate the association between multiple anthropometric and BIA-derived body composition parameters and IR as assessed by HOMA-IR and to evaluate the clinical utility of those parameters in prepubertal children with obesity.

## 2. Materials and Methods

### 2.1. Participants

This retrospective study was conducted at the Department of Pediatrics, Eunpyeong St. Mary’s Hospital, The Catholic University of Korea. We reviewed the medical records of prepubertal children with obesity who visited the pediatric endocrine clinic between 1 March 2020 and 28 February 2026.

The inclusion criteria were as follows: (1) girls aged 2 to <9 years and boys aged 2 to <10 years; (2) obesity, defined as a body mass index (BMI) ≥95th percentile for age and sex according to the 2017 Korean Children and Adolescents Growth Charts; and (3) prepubertal status, confirmed by Tanner stage 1 (no breast bud development in girls and testicular volume <4 mL in boys).

Subjects were excluded if they met any of the following criteria: (1) reached Tanner stage ≥2; (2) had underlying endocrine or metabolic disorders, such as precocious puberty, diabetes mellitus, thyroid disease, or Cushing’s syndrome; or (3) had insufficient anthropometric or body composition data for analysis.

The study protocol was approved by the Institutional Review Board (IRB) of Eunpyeong St. Mary’s Hospital, The Catholic University of Korea (IRB No. PC26RISI0079). Since this study was a retrospective chart review using de-identified data, the requirement for informed consent was waived by the IRB.

### 2.2. Anthropometric Measurements and Bone Age Assessment

Anthropometric parameters were measured by trained medical personnel using standardized protocols. To ensure accuracy, all anthropometric measurements were performed twice, and the average values were used for analysis. Height and weight were measured to the nearest 0.1 cm and 0.1 kg, respectively, using an automated stadiometer and scale (GL-300P; G-Tech International Co., Ltd., Uijeongbu, Republic of Korea). Body mass index (BMI) was calculated as weight divided by the square of height (kg/m^2^). Additionally, the obesity index (OI) was determined by calculating the percentage of the difference between the actual body weight and the standard weight for height, relative to the standard weight for height. Standard deviation scores (SDS) for height, weight, and BMI were determined based on the 2017 Korean growth charts [25].

Waist circumference (WC) was measured at the midpoint between the lower costal margin and the iliac crest at the end of a normal expiration, and WC_SDS was calculated. The waist-to-height ratio (WHtR) was calculated as WC (cm) divided by height (cm), and WC_SDS and WHtR_SDS were calculated by directly applying the age- and sex-specific L, M, and S parameters for Korean children and adolescents derived using the LMS method [26], according to the formula SDS = [(value/M^)L^ − 1]/(L × S).

Bone age (BA) was assessed from left-hand and wrist radiographs using the Greulich–Pyle (GP) method, and all radiographs were evaluated by a single experienced pediatric endocrinologist. The difference between bone age and chronological age (BA-CA) was calculated for each participant.

### 2.3. Body Composition Analysis

Body composition parameters were assessed using a multi-frequency, bioelectrical impedance analysis (BIA) device featuring an octopolar (8-electrode) method (InBody J50; InBody Co., Ltd., Seoul, Republic of Korea). This device utilizes multiple frequencies (5, 50, and 250 kHz) to ensure precise electrical impedance measurements. All BIA measurements were performed by trained pediatric nursing staff following a standardized departmental protocol. The device underwent routine calibration according to the manufacturer’s recommended schedule. To standardize hydration status, all measurements were performed in the morning after a minimum 4 h fast and immediately following voiding. Manufacturer-recommended quality-control procedures—including removal of metallic objects and outer clothing, bare-foot and bare-hand electrode contact, and standardized standing posture—were followed for every measurement. During the assessment, participants stood barefoot on the scale footplates (two electrode contact points per foot) and held a tactile handle in each hand (two electrode contact points per hand) for approximately 1 min. Each participant’s height, sex, and age were manually entered into the device prior to measurement. The directly measured variables include percent body fat (PBF, %), total fat mass (FM, kg), and skeletal muscle mass (SMM, kg). Based on these parameters, the fat-to-muscle ratio (FMR) was determined as the ratio of total fat mass to skeletal muscle mass [23]. In addition, the skeletal muscle index (SMI, %), calculated as the ratio of SMM to body weight multiplied by 100, was derived from the Inbody J50 output parameters to reflect the relative contribution of skeletal muscle mass to total body weight.

### 2.4. Biochemical Analysis and Insulin Resistance

Blood samples were collected after at least 8 h of fasting. Laboratory parameters included fasting blood sugar (FBS), HbA1c, fasting insulin, C-peptide, aspartate aminotransferase (AST), alanine aminotransferase (ALT), and a lipid profile (total cholesterol, triglycerides, HDL-C, and LDL-C). Thyroid function tests (T3, fT4, and TSH) were performed to screen for underlying thyroid disorders. Insulin resistance was evaluated using the homeostasis model assessment of insulin resistance (HOMA-IR) index. The HOMA-IR value was determined as the product of fasting insulin (uIU/mL) and fasting glucose (mg/dL), divided by 405. For the purposes of this study, insulin resistance (IR) was defined as a HOMA-IR value equal to or greater than 2.5, which falls within the ROC-derived cutoff range of 2.22–2.67 reported for prepubertal obese children [27] and is consistent with thresholds widely applied in pediatric obesity research [28,29].

### 2.5. Statistical Analysis

Statistical analyses were performed using IBM SPSS Statistics for Windows, version 21.0 (IBM Corp., Armonk, NY, USA). Continuous variables are expressed as mean ± standard deviation (SD), and categorical variables are presented as frequencies and percentages (*n*, %). The normality of the data distribution was assessed using the Kolmogorov–Smirnov test.

An independent-sample *t*-test was used for normally distributed continuous variables, and the Mann–Whitney U test was performed for non-normally distributed variables. Categorical variables were compared using the chi-square test or Fisher’s exact test, as appropriate.

Pearson’s or Spearman’s correlation analysis was used to evaluate the association between body composition indices (BMI_SDS, WC_SDS, PBF, and FMR) and HOMA-IR, depending on the distribution of variables. Because no universally validated HOMA-IR cutoff exists for prepubertal children, insulin resistance was analyzed both as a continuous variable and as a binary outcome. To identify the independent predictors of HOMA-IR, multiple linear regression analysis was performed with HOMA-IR as the continuous outcome variable. To identify independent risk factors for insulin resistance, multivariate logistic regression analysis was conducted with HOMA-IR ≥ as the binary outcome variable. Both regression models were adjusted for age and sex. Results of the logistic regression are presented as odds ratios (ORs) with 95% confidence intervals (CIs). A two-sided *p*-value of <0.05 was considered statistically significant for all analyses.

## 3. Results

### 3.1. Baseline Characteristics of the Study Population

A total of 147 prepubertal children with obesity were enrolled in this study, comprising 61 males (41.5%) and 86 females (58.5%). The baseline clinical and anthropometric characteristics of the entire cohort are summarized in Table 1. The mean age was 7.40 ± 1.00 years, and the mean bone age was 9.58 ± 1.51 years. The mean BMI SDS and obesity index were 2.58 ± 0.86 and 136.8 ± 12.1%, respectively. The mean percent body fat (PBF) was 38.44 ± 5.13%, and the mean fat-to-muscle ratio (FMR) was 1.25 ± 0.27. The mean HOMA-IR for the cohort was 2.94 ± 1.79.

### 3.2. Comparison of Clinical and Metabolic Parameters According to Insulin Resistance Status

Subjects were classified into two groups based on HOMA-IR: the non-IR group (HOMA-IR < 2.5; *n* = 66) and the IR group (HOMA-IR ≥ 2.5; *n* = 72). Fasting insulin and C-peptide values, required for HOMA-IR calculation, were unavailable for 9 of the 147 enrolled participants; these participants were therefore excluded from analyses stratified by IR status. Comparisons of clinical and biochemical parameters between the two groups are presented in Table 2. Sex distribution did not differ significantly between groups (*p* = 0.799). Although age showed only a marginal trend (7.24 ± 1.02 vs. 7.54 ± 0.95 years; *p* = 0.085), bone age (9.16 ± 1.57 vs. 9.97 ± 1.42 years; *p* = 0.002) and bone age advancement (BA − CA: 1.91 ± 1.33 vs. 2.46 ± 1.31 years; *p* = 0.017) were significantly greater in the IR group.

All adiposity-related anthropometric parameters were significantly higher in the IR group, including BMI SDS (2.34 ± 0.90 vs. 2.79 ± 0.80; *p* = 0.015), obesity index (133.0 ± 9.9% vs. 141.3 ± 12.7%; *p* < 0.001), WC SDS (2.50 ± 0.46 vs. 2.81 ± 0.55; *p* < 0.001), WHtR SDS (2.57 ± 0.52 vs. 2.89 ± 0.50; *p* < 0.001), and PBF (37.21 ± 5.03% vs. 39.87 ± 5.06%; *p* = 0.002). FMR was also significantly elevated in the IR group (1.20 ± 0.24 vs. 1.30 ± 0.30; *p* = 0.035), whereas SMI% did not differ significantly between groups (31.70 ± 2.50% vs. 31.40 ± 4.35%; *p* = 0.630).

Regarding glycemic parameters, fasting glucose (92.7 ± 5.4 vs. 95.0 ± 5.6 mg/dL; *p* = 0.013), HbA1c (5.43 ± 0.07% vs. 5.54 ± 0.25%; *p* = 0.010), fasting insulin (7.41 ± 2.26 vs. 17.37 ± 7.63 μIU/mL; *p* < 0.001), and C-peptide (1.62 ± 1.01 vs. 2.83 ± 1.08 ng/mL; *p* < 0.001) were all significantly higher in the IR group. Among lipid parameters, triglycerides were significantly elevated (79.6 ± 37.0 vs. 96.5 ± 51.7 mg/dL; *p* = 0.031), and HDL cholesterol was significantly reduced (54.5 ± 10.4 vs. 50.6 ± 12.2 mg/dL; *p* = 0.047) in the IR group. ALT was significantly higher in the IR group (19.2 ± 10.9 vs. 32.6 ± 39.0 IU/L; *p* = 0.008). Total cholesterol, LDL cholesterol, AST, and thyroid function tests did not differ significantly between groups.

### 3.3. Correlation and Linear Regression Analysis of Factors Associated with HOMA-IR

**Figure 1:**
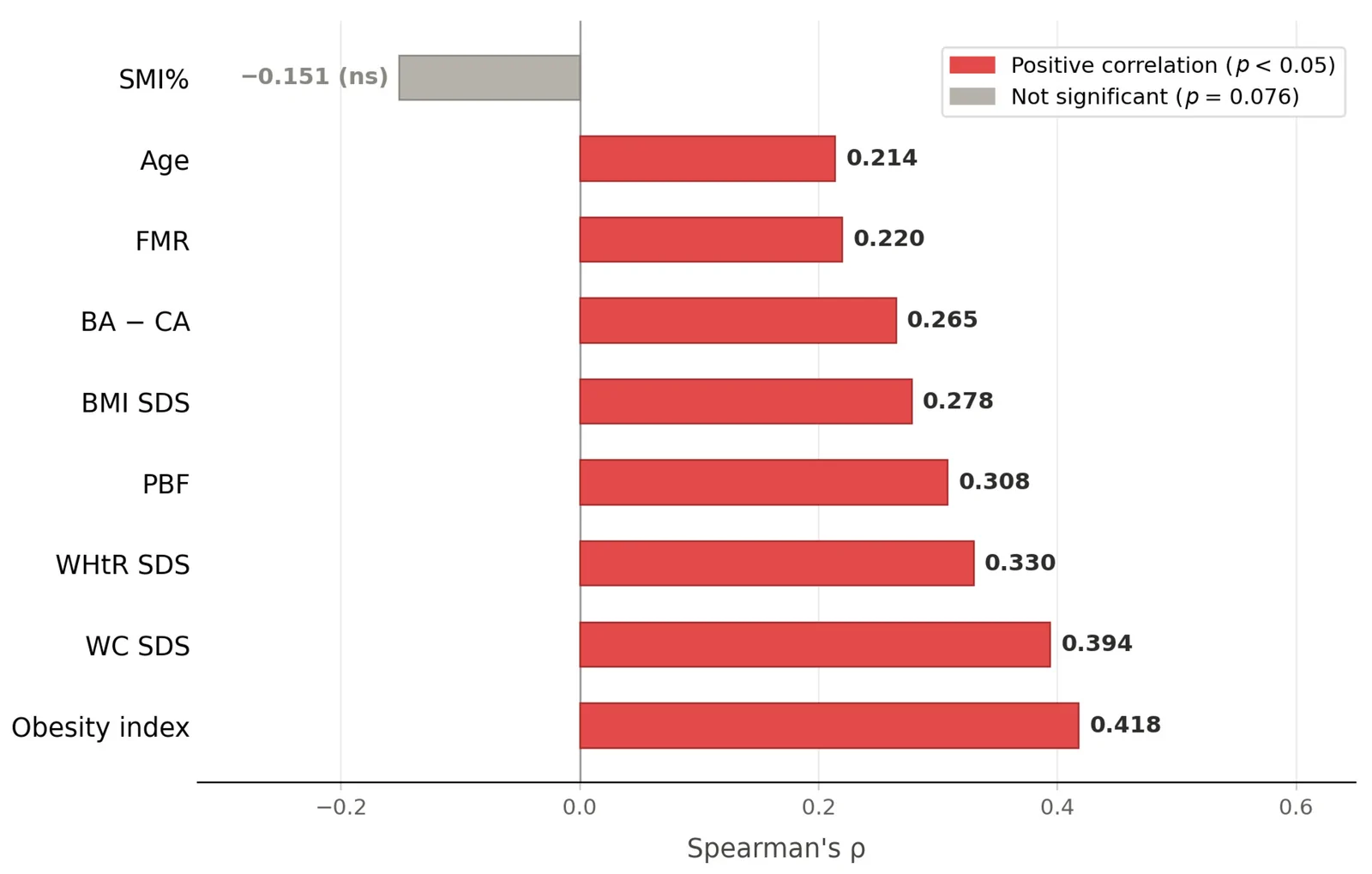
Spearman correlation coefficients between HOMA-IR and clinical parameters in prepubertal children with obesity. Red bars indicate statistically significant positive correlations (*p* < 0.05). The gray bar (SMI%) indicates a non-significant trend toward negative correlation (ρ = −0.151; *p* = 0.076). Variables are ordered by effect size. Abbreviations: BA − CA, bone age minus chronological age; BMI SDS, body mass index standard deviation score; FMR, fat-to-muscle ratio; HOMA-IR, homeostatic model assessment for insulin resistance; PBF, percent body fat; SMI%, skeletal muscle index; WC SDS, waist circumference standard deviation score; WHtR SDS, waist-to-height ratio standard deviation score.

Given the non-normal distribution of key variables confirmed by normality testing, Spearman rank correlation analysis was performed to assess associations between HOMA-IR and clinical parameters (Figure 1). Significant positive correlations with HOMA-IR were identified for WC SDS (ρ = 0.39; *p* < 0.001), obesity index (ρ = 0.42; *p* < 0.001), WHtR SDS (ρ = 0.33; *p* < 0.001), PBF (ρ = 0.31; *p* < 0.001), BMI SDS (ρ = 0.28; *p* < 0.001), BA − CA (ρ = 0.27; *p* = 0.002), FMR (ρ = 0.22; *p* = 0.006), and age (ρ = 0.21; *p* = 0.012). SMI% showed a trend toward a negative correlation with HOMA-IR (ρ = −0.15; *p* = 0.076) that did not reach statistical significance.

In univariate linear regression analysis, age (β = 0.35; *p* = 0.024), BMI SDS (β = 0.57; *p* = 0.001), WC SDS (β = 1.35; *p* < 0.001), PBF (β = 0.12; *p* < 0.001), and FMR (β = 1.72; *p* = 0.001) were each significantly associated with HOMA-IR (Table 3). In multivariate linear regression adjusted for age and sex, BMI SDS (β = 0.70; *p* < 0.001), WC SDS (β = 1.92; *p* < 0.001), PBF (β = 0.12; *p* < 0.001), and FMR (β = 1.81; *p* < 0.001) remained independent predictors of HOMA-IR.

### 3.4. Independent Risk Factors for Insulin Resistance

Logistic regression analysis was performed to identify independent risk factors for IR (HOMA-IR ≥ 2.5; Table 4). In univariate analysis, BMI SDS (OR = 1.899; 95% CI: 1.105–3.263; *p* = 0.002), WC SDS (OR = 3.590; 95% CI: 1.626–7.571; *p* = 0.001), PBF (OR = 1.115; 95% CI: 1.010–1.201; *p* = 0.004), and FMR (OR = 3.946; 95% CI: 1.080–14.420; *p* = 0.038) were significant predictors of IR, whereas age and sex were not (*p* = 0.094 and *p* = 0.799, respectively).

In multivariate logistic regression adjusted for age and sex, WC SDS (OR = 6.040; 95% CI: 2.470–14.752; *p* < 0.001), FMR (OR = 4.409; 95% CI: 1.158–16.781; *p* = 0.030), BMI SDS (OR = 2.309; 95% CI: 1.299–4.104; *p* = 0.004), and PBF (OR = 1.115; 95% CI: 1.035–1.203; *p* = 0.004) remained independent risk factors for IR. Notably, central adiposity indices, including BMI SDS, PBF, and FMR, were each independently associated with IR, suggesting that multiple distinct adiposity compartments contribute to the development of IR in prepubertal children with obesity.

In additional exploratory analyses, BRI and CMI were also independently associated with IR after adjustment for age and sex, whereas ABSI and the TyG index were not; as no pediatric reference values permitting SDS transformation exist for these indices, they were analyzed as raw values, and detailed results are provided in Supplementary Table S1.

### 3.5. ROC Curve Analysis for Predicting Insulin Resistance

To evaluate the discriminatory performance of the anthropometric and body composition indices for identifying IR, ROC curve analysis was performed (Table 5). WC SDS demonstrated the highest discriminatory ability (AUC = 0.685; 95% CI: 0.586–0.778; *p* < 0.001), with an optimal cutoff of 2.74 yielding a sensitivity of 58.7% and specificity of 72.6%. BMI SDS (AUC = 0.670), PBF (AUC = 0.640), and FMR (AUC = 0.602) also showed statistically significant, though more modest, discriminatory performance.

## 4. Discussion

The present study investigated the associations between body composition parameters and insulin resistance (IR) in prepubertal children with obesity. In multivariable logistic regression, WC SDS, FMR, BMI SDS, and PBF were each significantly associated with IR after adjustment for age and sex. These findings indicate that central adiposity and the balance between fat and muscle mass are relevant to metabolic risk in this population, complementing rather than replacing generalized obesity measures. Notably, all subjects were confirmed to be at Tanner stage I, effectively eliminating the confounding influence of puberty-induced physiological IR—a crucial methodological strength, given that insulin sensitivity is known to decline substantially during pubertal transition and may independently alter both fat distribution and metabolic risk [5,24].

The identification of WC SDS as a significant independent predictor of IR is consistent with prior evidence demonstrating that central adiposity is a more potent metabolic risk factor than global adiposity in the pediatric age group [5,13,15]. BMI, while widely used, reflects overall body mass without distinguishing between subcutaneous and visceral adipose tissue depots and has been shown to be a limited indicator of cardiometabolic risk at the individual level [8,14]. In contrast, WC serves as an established surrogate for abdominal adiposity [8,30]. Visceral adipose tissue is highly metabolically active and promotes IR through the release of free fatty acids and pro-inflammatory adipokines—including TNF-α and IL-6—directly into the portal circulation, thereby impairing insulin signaling in the liver and peripheral tissues [5]. Recent evidence further supports the complementary use of WC or WHtR alongside BMI in the clinical assessment of obesity-related metabolic risk [8,14]. Consistent with prior population-based data from Korean children and adolescents demonstrating a steep rise in IR prevalence with increasing obesity class [4], our cohort—comprising exclusively prepubertal children with obesity—showed a notably high IR prevalence, underscoring the early metabolic burden of obesity even before the onset of puberty.

A further finding of this study is that FMR was significantly associated with IR after adjustment for age and sex (OR = 4.41; 95% CI 1.16–16.78; *p* = 0.030). Skeletal muscle is the primary site of insulin-stimulated glucose disposal, accounting for the majority of postprandial glucose uptake; accordingly, an imbalance between excess fat and relatively reduced muscle mass is expected to impair whole-body insulin sensitivity [5,23]. While PBF captures only the proportion of body fat, FMR provides a composite index reflecting this fat-to-muscle balance [23,31]. The independent significance of FMR beyond PBF (OR = 1.115 per 1% increment) suggests that the relative imbalance between fat and muscle—rather than fat excess alone—is a more meaningful determinant of IR risk in this population. This finding extends prior observations in adult populations—where higher FMR has been independently associated with metabolic syndrome and IR [22,23]—to the prepubertal age group, suggesting that the metabolic relevance of fat-to-muscle imbalance may originate well before adolescence. Importantly, SMI% did not significantly differ between the IR and non-IR groups (31.70% vs. 31.40%; *p* = 0.630) and showed only a non-significant trend toward negative correlation with HOMA-IR (ρ = −0.151; *p* = 0.076). This pattern is consistent with prior meta-analytic evidence demonstrating that lean body mass percentage is modestly lower in children with IR, whereas absolute lean mass tends to be higher due to body size effects. Taken together, these findings suggest that it is the excess of fat relative to muscle—captured by FMR—rather than absolute muscle deficiency per se that confers metabolic risk in obese prepubertal children [32].

The IR group also exhibited significantly greater bone age advancement relative to chronological age (BA − CA: 2.46 ± 1.31 vs. 1.91 ± 1.33 years; *p* = 0.017), and BA − CA showed a significant positive correlation with HOMA-IR (ρ = 0.265; *p* = 0.002). The precise mechanism underlying this association warrants further investigation but may involve the anabolic effects of chronically elevated insulin on skeletal maturation pathways. Given that hyperinsulinemia-driven growth acceleration may also manifest as increased linear height, we further examined Height SDS—although not included as an independent predictor because of collinearity with WC SDS and BMI SDS—as a potential confounder of the primary associations. Height SDS did not differ significantly between the non-IR and IR groups (0.91 ± 0.94 vs. 1.23 ± 1.08; *p* = 0.058), and additional adjustment for Height SDS in sensitivity analyses did not materially change the associations of WC SDS, BMI SDS, PBF, or FMR with IR (Supplementary Table S2), indicating that linear growth status does not meaningfully confound the reported associations. Furthermore, the IR group demonstrated a clustering of early metabolic syndrome components [23,33], including significantly elevated triglycerides (*p* = 0.031), reduced HDL cholesterol (*p* = 0.047), and elevated ALT (*p* = 0.008), suggesting early-onset hepatic IR and dyslipidemia in this prepubertal cohort [33,34]. These findings underscore the clinical importance of early and comprehensive metabolic screening in obese prepubertal children, as the window for preventive intervention may precede the onset of overt dyslipidemia and hepatic injury.

Our findings should be interpreted in the context of the existing pediatric literature in which waist circumference has repeatedly been shown to relate to insulin resistance. A previous study reported that WC predicted HOMA-IR–defined insulin resistance in schoolchildren aged 6–13 years and proposed its use as a simple office-based screening tool [35], and subsequent reviews have advocated a two-step strategy in which BMI serves for initial screening and WC or WHtR for the assessment of central adiposity [14]. The present study extends this body of work in three respects. First, prior pediatric studies have generally enrolled participants spanning a broad age range and multiple Tanner stages, whereas puberty itself induces a substantial, adiposity-independent decline in insulin sensitivity [24]; by restricting the cohort to children with confirmed Tanner stage 1, we demonstrate that the association between central adiposity and IR is already established before any pubertal contribution. Second, whereas earlier studies relied predominantly on tape-measure anthropometry, we incorporated BIA-derived body composition and found that FMR remained independently associated with IR in age- and sex-adjusted analysis (OR = 4.41; 95% CI 1.16–16.78; *p* = 0.030), indicating that the qualitative fat–muscle imbalance previously characterized mainly in adults [22,23] is already detectable in the prepubertal period. Third, by evaluating conventional anthropometric and BIA-derived indices within a single, pubertal-stage-homogeneous cohort under a uniform analytical framework, we provide a consistent description of how these indices relate to IR in this specific age group.

The present findings have important clinical implications. Current pediatric obesity assessment relies predominantly on BMI; however, a landmark international commission published in 2025 formally recommended moving beyond BMI alone, advocating for complementary measurement of at least one additional indicator of fat distribution—such as WC or waist-to-height ratio—to more accurately capture individual-level cardiometabolic risk [14]. Our data provide pediatric-specific support for this recommendation. From a practical standpoint, both WC and BIA-derived FMR are readily obtainable in routine outpatient settings: WC requires only a standard measuring tape, and BIA devices—increasingly available in pediatric obesity clinics—provide FMR as part of standard output [8]. Furthermore, the significant association of WC and FMR with IR in our age- and sex-adjusted models raises the possibility that children with relatively lower BMI but elevated WC or FMR may represent an underrecognized high-risk subgroup—analogous to the so-called “metabolically obese normal-weight” phenotype—warranting prospective investigation in future studies [14].

To further contextualize the clinical utility of these indices, we performed ROC curve analysis for WC SDS, BMI SDS, PBF, and FMR (Table 5). The AUC values observed (0.60–0.69) indicate fair, rather than excellent, discriminatory performance for any single index, reflecting the multifactorial pathogenesis of pediatric insulin resistance. These indices are therefore best applied as complementary components of a comprehensive clinical risk assessment rather than as stand-alone diagnostic tools.

This study has several limitations that warrant consideration. First, the cross-sectional design precludes the establishment of causal relationships, and longitudinal studies are required to confirm whether elevated WC SDS and FMR prospectively predict incident IR, type 2 diabetes, or cardiovascular disease as these children progress through puberty and into adulthood. Second, the single-center design and relatively modest sample size may limit the generalizability of the findings. Third, while HOMA-IR is the most widely used surrogate measure of IR in epidemiological settings [24], no universally validated cutoff exists for IR in prepubertal children. The threshold of HOMA-IR ≥ 2.5 applied in this study was informed by previous studies reporting ROC-derived cutoffs of 2.22–2.67 in prepubertal children with obesity [27,29]. Nevertheless, results should be interpreted with caution given the absence of a population-specific validated cutoff for Korean prepubertal children. Fourth, body composition was assessed using BIA rather than DXA; although BIA is well-validated and practical for clinical use, it does not provide information on regional fat distribution or ectopic fat depots—particularly hepatic and visceral fat—which are mechanistically relevant to IR pathogenesis [5]. Fifth, adipocytokines (e.g., leptin, adiponectin) and growth hormone–IGF-1 axis parameters were not measured; incorporating these biomarkers in future prospective studies may further clarify the hormonal mechanisms linking body composition to insulin resistance in this population.

## 5. Conclusions

WC SDS and FMR are each independent predictors of IR in prepubertal children with obesity. These findings support a paradigm shift in the clinical evaluation of pediatric obesity—from reliance on generalized measures of adiposity to integrated assessment of central adiposity and fat-to-muscle balance—to facilitate earlier identification of children at highest metabolic risk.

## Figures and Tables

**Table 1:** Baseline characteristics of prepubertal children with obesity.

Variable	Total (*n* = 147)	Male (*n* = 61)	Female (*n* = 86)	*p*-Value
Demographic and auxological parameters				
Age (years)	7.40 ± 1.00	7.90 ± 0.94	7.05 ± 0.88	<0.001
Bone age (years)	9.58 ± 1.51	9.48 ± 1.84	9.65 ± 1.23	0.495
BA − CA (years)	2.18 ± 1.32	1.58 ± 1.49	2.61 ± 1.00	<0.001
Height SDS	1.04 ± 1.02	0.95 ± 0.97	1.11 ± 1.05	0.498
Weight SDS	2.23 ± 0.71	2.18 ± 0.71	2.26 ± 0.71	0.518
BMI SDS	2.58 ± 0.86	2.53 ± 0.73	2.61 ± 0.95	0.641
Obesity index (%)	136.8 ± 12.1	137.5 ± 13.5	136.4 ± 11.0	0.900
WC SDS	2.63 ± 0.54	2.40 ± 0.54	2.80 ± 0.48	<0.001
WHtR SDS	2.71 ± 0.55	2.54 ± 0.53	2.84 ± 0.52	0.002
Body composition parameters (BIA)				
PBF (%)	38.44 ± 5.13	38.72 ± 5.37	38.25 ± 4.98	0.920
SMI% (%)	31.59 ± 3.49	32.20 ± 4.56	31.16 ± 2.40	0.061
FMR	1.25 ± 0.27	1.24 ± 0.32	1.25 ± 0.24	0.315
Glycemic parameters				
Fasting glucose (mg/dL)	94.1 ± 5.9	95.7 ± 5.9	92.9 ± 5.6	0.009
HbA1c (%)	5.48 ± 0.26	5.50 ± 0.27	5.47 ± 0.26	0.624
Insulin (μIU/mL)	12.60 ± 7.59	13.33 ± 9.06	12.08 ± 6.32	0.615
C-peptide (ng/mL)	2.25 ± 1.21	2.24 ± 1.19	2.25 ± 1.22	0.725
HOMA-IR	2.94 ± 1.79	3.15 ± 2.11	2.79 ± 1.51	0.434
Lipid parameters				
Total cholesterol (mg/dL)	180.0 ± 29.3	180.3 ± 30.6	179.8 ± 28.5	0.945
Triglycerides (mg/dL)	87.6 ± 45.2	78.8 ± 35.6	93.8 ± 50.1	0.092
HDL-C (mg/dL)	52.3 ± 11.4	53.1 ± 12.3	51.8 ± 10.8	0.434
LDL-C (mg/dL)	109.4 ± 25.6	108.0 ± 25.1	110.4 ± 26.2	0.474
Hepatic parameters				
AST (IU/L)	29.4 ± 9.0	30.6 ± 12.1	28.6 ± 5.9	0.619
ALT (IU/L)	25.8 ± 29.2	33.0 ± 41.8	20.7 ± 12.7	0.001
Thyroid function				
T3 (ng/mL)	1.71 ± 0.24	1.72 ± 0.34	1.70 ± 0.17	0.715
Free T4 (ng/dL)	1.29 ± 0.13	1.28 ± 0.16	1.30 ± 0.11	0.631
TSH (mIU/L)	2.91 ± 1.71	2.93 ± 1.76	2.89 ± 1.68	0.898

Abbreviations: ALT, alanine aminotransferase; AST, aspartate aminotransferase; BA − CA, bone age minus chronological age; BIA, bioelectrical impedance analysis; BMI SDS, body mass index standard deviation score; FMR, fat-to-muscle ratio; HbA1c, glycated hemoglobin; HDL-C, high-density lipoprotein cholesterol; HOMA-IR, homeostatic model assessment for insulin resistance; LDL-C, low-density lipoprotein cholesterol; PBF, percent body fat; SDS, standard deviation score; SMI%, skeletal muscle index; T3, triiodothyronine; free T4, free thyroxine; TSH, thyroid-stimulating hormone; WC SDS, waist circumference standard deviation score; WHtR SDS, waist-to-height ratio standard deviation score. Values are expressed as mean ± standard deviation. *p*-values compare males and females and were calculated using the Mann–Whitney U test.

**Table 2:** Comparison of clinical and biochemical parameters in prepubertal children with obesity according to insulin resistance status.

Variable	HOMA-IR < 2.5 (*n* = 66)	HOMA-IR ≥ 2.5 (*n* = 72)	*p*-Value
Demographic and auxological parameters			
Sex, male	27 (40.9%)	31 (43.1%)	0.799
Age (years)	7.24 ± 1.02	7.54 ± 0.95	0.085
Bone age (years)	9.16 ± 1.57	9.97 ± 1.42	0.002
BA − CA (years)	1.91 ± 1.33	2.46 ± 1.31	0.017
BMI SDS	2.34 ± 0.90	2.79 ± 0.80	0.015
Obesity index (%)	133.0 ± 9.9	141.3 ± 12.7	<0.001
WC SDS	2.50 ± 0.46	2.81 ± 0.55	<0.001
WHtR SDS	2.57 ± 0.52	2.89 ± 0.50	<0.001
Body composition parameters (BIA)			
PBF (%)	37.21 ± 5.03	39.87 ± 5.06	0.002
SMI% (%)	31.70 ± 2.50	31.40 ± 4.35	0.630
FMR	1.20 ± 0.24	1.30 ± 0.30	0.035
Glycemic parameters			
Fasting glucose (mg/dL)	92.7 ± 5.4	95.0 ± 5.6	0.013
HbA1c (%)	5.43 ± 0.07	5.54 ± 0.25	0.010
Insulin (μIU/mL)	7.41 ± 2.26	17.37 ± 7.63	<0.001
C-peptide (ng/mL)	1.62 ± 1.01	2.83 ± 1.08	<0.001
Lipid parameters			
Total cholesterol (mg/dL)	184.1 ± 29.6	176.7 ± 29.5	0.414
Triglycerides (mg/dL)	79.6 ± 37.0	96.5 ± 51.7	0.031
HDL-C (mg/dL)	54.5 ± 10.4	50.6 ± 12.2	0.047
LDL-C (mg/dL)	111.6 ± 25.2	107.7 ± 26.3	0.385
Hepatic parameters			
AST (IU/L)	28.4 ± 4.6	30.3 ± 11.5	0.221
ALT (IU/L)	19.2 ± 10.9	32.6 ± 39.0	0.008
Thyroid function			
T3 (ng/mL)	1.68 ± 0.14	1.76 ± 0.31	0.102
Free T4 (ng/dL)	1.31 ± 0.11	1.28 ± 0.15	0.235
TSH (mIU/L)	3.23 ± 2.14	2.70 ± 1.20	0.073

Abbreviations: ALT, alanine aminotransferase; AST, aspartate aminotransferase; BA − CA, bone age minus chronological age; BMI SDS, body mass index standard deviation score; FMR, fat-to-muscle ratio; HDL-C, high-density lipoprotein cholesterol; HbA1c, glycated hemoglobin; HOMA-IR, homeostatic model assessment for insulin resistance; IR, insulin resistance; LDL-C, low-density lipoprotein cholesterol; PBF, percent body fat; SMI%, skeletal muscle index; T3, triiodothyronine; free T4, free thyroxine; TSH, thyroid-stimulating hormone; WC SDS, waist circumference standard deviation score; WHtR SDS, waist-to-height ratio standard deviation score. Values are expressed as mean ± standard deviation or *n* (%). IR was defined as HOMA-IR ≥ 2.5.

**Table 3:** Linear regression analysis of factors associated with HOMA-IR.

Risk Factor	Univariate			Multivariate †		
	β	SE	*p*-Value	β	SE	*p*-Value
Age	0.35	0.15	0.024	—	—	—
Sex	0.36	0.31	0.241	—	—	—
BMI SDS	0.57	0.171	0.001	0.70	0.17	<0.001
WC SDS	1.35	0.29	<0.001	1.92	0.28	<0.001
PBF (%)	0.12	0.04	<0.001	0.13	0.03	<0.001
FMR	1.72	0.54	0.001	1.81	0.53	<0.001

Abbreviations: BMI SDS, body mass index standard deviation score; FMR, fat-to-muscle ratio; HOMA-IR, homeostatic model assessment for insulin resistance; PBF, percent body fat; SE, standard error; WC SDS, waist circumference standard deviation score. † Multivariate analysis was adjusted for age and sex.

**Table 4:** Multivariate logistic regression analysis of risk factors for insulin resistance.

Risk Factor	Univariate			Multivariate †		
	OR	95% CI	*p*-Value	OR	95% CI	*p*-Value
Age	1.35	0.95–1.91	0.094	—	—	—
Sex	1.09	0.56–2.15	0.799	—	—	—
BMI SDS	1.90	1.11–3.26	0.020	2.31	1.30–4.10	0.004
WC SDS	3.51	1.63–7.57	0.001	6.04	2.47–14.75	<0.001
PBF (%)	1.12	1.01–1.20	0.004	1.12	1.04–1.20	0.004
FMR	3.95	1.08–14.42	0.038	4.41	1.16–16.78	0.030

Abbreviations: BMI SDS, body mass index standard deviation score; CI, confidence interval; FMR, fat-to-muscle ratio; OR, odds ratio; PBF, percent body fat; WC SDS, waist circumference standard deviation score. † Multivariate analysis was adjusted for age and sex. IR was defined as HOMA-IR ≥2.5.

**Table 5:** Receiver operating characteristic (ROC) curve analysis of body composition indices for predicting insulin resistance.

Variable	AUC (95% CI)	*p*-Value	Optimal Cutoff	Sensitivity (%)	Specificity (%)
WC SDS	0.685 (0.586–0.778)	<0.001	≥2.74	58.7	72.6
BMI SDS	0.670 (0.572–0.759)	<0.001	≥2.55	59.7	68.2
PBF (%)	0.640 (0.550–0.729)	0.004	≥40.2	45.8	77.3
FMR	0.602 (0.505–0.692)	0.039	≥1.12	76.4	45.5

Abbreviations: AUC, area under the curve; BMI SDS, body mass index standard deviation score; CI, confidence interval; FMR, fat-to-muscle ratio; PBF, percent body fat; WC SDS, waist circumference standard deviation score. Optimal cutoffs were determined using the Youden index. IR was defined as HOMA-IR ≥2.5.

## Data Availability

The data presented in this study are not publicly available due to privacy and ethical restrictions governing the use of patient medical records. Data are available from the corresponding author upon reasonable request and subject to institutional data governance policies.

## Supplementary Materials

The following supporting information can be downloaded at https://www.mdpi.com/article/10.3390/metabo16080569/s1, Table S1: Association of ABSI, BRI, CMI, and TyG index with HOMA-IR and body composition parameters; Table S2: Sensitivity analysis of the independent risk factors for insulin resistance after additional adjustment for Height SDS.

## Abbreviations

The following abbreviations are used in this manuscript:

ALT	Alanine aminotransferase
AST	Aspartate aminotransferase
BA	Bone age
BA-CA	Bone age minus chronological age
BIA	Bioelectrical impedance analysis
BRI	Body roundness index
BMI	Body mass index
CMI	Cardiometabolic index
FBS	Fasting blood sugar
FM	Fat mass
FMR	Fat-to-muscle ratio
GP	Greulich–Pyle
HOMA-IR	Homeostasis model assessment of insulin resistance
IR	Insulin resistance
OI	Obesity index
PBF	Percent body fat
SDS	Standard deviation score
SMI%	Skeletal muscle index
TyG	The triglyceride–glucose index
WC	Waist circumference
WHtR	Waist-to-height ratio
