# Supplementary material for: Association of Waist Circumference and Body Composition Indices with Insulin Resistance in Prepubertal Children with Obesity
Source: Metabolites. 2026 Aug 11;16(8):569. doi: 10.3390/metabo16080569 (PMC13515845; doi:10.3390/metabo16080569)
Supplement: Supplementary file 1 [file metabolites-16-00569-s001.zip › metabolites-4436911-supplementary.pdf]

Supplementary

Table S1. Association of ABSI, BRI, CMI, and TyG index with HOMA-IR and body composition parameters.

**Index**	**ρ with HOMA-IR (P)**	**ρ with PBF (P)**	**ρ with FMR (P)**	**Adjusted OR* (95% CI), P**
ABSI	0.00 (0.998)	0.09 (0.312)	0.09 (0.316)	0.91 (0.63–1.31), P = 0.601
BRI	0.39 (P < 0.001)	0.68 (P < 0.001)	0.66 (P < 0.001)	2.28 (1.46–3.55), P < 0.001
CMI	0.27 (0.003)	0.18 (0.045)	0.21 (0.018)	1.76 (1.15–2.70), P = 0.010
TyG index	0.28 (0.001)	0.04 (0.649)	0.07 (0.419)	1.42 (0.98–2.05), P = 0.062

*Values are Spearman's rank correlation coefficients (ρ) given the non-normal distribution of HOMA-IR, consistent with the primary correlation analysis. *Adjusted for age and sex; odds ratios (OR) are expressed per 1-SD increase in each index, with the binary outcome HOMA-IR ≥ 2.5 defining insulin resistance (IR). n = 125 (ABSI, BRI) and n = 124 (CMI) for analyses involving HOMA-IR/IR status; n = 132 (ABSI, BRI) and n = 130 (CMI) for correlations with PBF/FMR; n = 137 (TyG vs. HOMA-IR/IR) and n = 143 (TyG vs. PBF/FMR), reflecting missing waist circumference or lipid data in a subset of participants. ABSI = $WC / (BMI^{2/3} \times height^{1/2})$; BRI = $364.2 - 365.5 \times \sqrt{[1 - (WC/2\pi)^2 / (0.5 \times height)^2]}$; CMI = WHtR × TG/HDL-C; TyG index = ln[fasting TG (mg/dL) × fasting glucose (mg/dL)/2]. Abbreviations as defined in the manuscript.*

Table S2. Sensitivity analysis of the independent risk factors for insulin resistance after additional adjustment for Height SDS.

**Variable**	**Original model (age+sex-adjusted) OR (95% CI), P**	**+Height SDS-adjusted OR (95% CI), P**	**Height SDS in this model: OR (95% CI), P**
WC SDS	6.04 (2.47–14.75), <0.001	6.58 (2.28–19.02), <0.001	0.93 (0.59–1.47), 0.763
BMI SDS	2.31 (1.30–4.10), 0.004	2.10 (1.15–3.82), 0.015	1.24 (0.86–1.80), 0.252
PBF (%)	1.12 (1.04–1.20), 0.004	1.11 (1.03–1.20), 0.006	1.40 (0.98–2.00), 0.065
FMR	4.41 (1.16–16.78), 0.030	4.54 (1.18–17.51), 0.028	1.44 (1.01–2.05), 0.043

Original model adjusted for age and sex, consistent with Table 4 of the main manuscript; Height SDS-adjusted model additionally adjusted for Height SDS. IR was defined as HOMA-IR ≥ 2.5. Abbreviations as defined in the manuscript.
